# Epstein-Barr Virus-Induced Hemophagocytic Lymphohistiocytosis: A Case Report of a Rare and Life-Threatening Syndrome

**DOI:** 10.7759/cureus.76604

**Published:** 2024-12-30

**Authors:** Usamah Al-Anbagi, Abdulqadir J Nashwan, Claret Charles Isabirye

**Affiliations:** 1 Internal Medicine Department, Hamad Medical Corporation, Doha, QAT; 2 Nursing & Midwifery Research Department, Hamad Medical Corporation, Doha, QAT

**Keywords:** allogeneic hematopoietic cell transplant (hct), bone marrow biopsy, cytopenia, epstein-barr virus (ebv), etoposide, ferritin, hemophagocytic lymphohistiocytosis (hlh), hypertriglyceridemia

## Abstract

Hemophagocytic lymphohistiocytosis (HLH) is a rare, life-threatening syndrome of excessive immune activation, leading to severe inflammation and organ damage. While more common in infants, HLH can occur at any age and is often triggered by infections such as Epstein-Barr virus (EBV). In this case, a 38-year-old man presented with a three-week history of fevers, night sweats, poor appetite, and severe anemia. Investigations revealed hepatosplenomegaly, extremely elevated ferritin levels, triglycerides, and a positive EBV PCR. Despite treatment, his condition deteriorated, and a bone marrow biopsy confirmed HLH. He received immunosuppressive therapy but ultimately passed away after 64 days in the hospital. This case emphasizes the diagnostic challenges of HLH, particularly when triggered by the EBV. Early diagnosis and prompt treatment are vital, although prognosis can be poor in severe cases, underscoring the importance of clinical vigilance.

## Introduction

Hemophagocytic lymphohistiocytosis (HLH) is a severe and potentially fatal syndrome characterized by an uncontrolled immune response that leads to excessive inflammation and tissue destruction. This hyper-inflammatory condition can arise from genetic defects or as a secondary phenomenon triggered by various insults, including infections, autoimmune diseases, and malignancies [1,2]. Although HLH predominantly affects infants and young children, it is increasingly recognized in adults, where its clinical manifestations may be more varied and complex, often complicating timely diagnosis [3].

The pathophysiology of HLH involves a dysregulated immune system, wherein activated macrophages and lymphocytes fail to downregulate appropriately, resulting in the overproduction of inflammatory cytokines such as tumor necrosis factor-alpha and interferon-gamma. Epstein-Barr virus (EBV) is one of the most significant infectious agents associated with secondary HLH, frequently complicating cases in both pediatric and adult populations [4]. Patients typically present with a constellation of symptoms, including persistent fever, hepatosplenomegaly, cytopenias, and hypertriglyceridemia, which can mimic other infectious or inflammatory conditions, leading to delays in diagnosis [5].

Prompt recognition and intervention are crucial due to the high morbidity and mortality associated with HLH. Diagnosis is generally guided by well-established clinical criteria, augmented by laboratory findings indicative of immune dysregulation [6]. This case report details a 38-year-old male who presented with acute symptoms suggestive of HLH, including marked cytopenias and elevated inflammatory markers. Despite an initial misdiagnosis, further investigations revealed EBV as the underlying trigger for his condition. This case underscores the importance of considering HLH in differential diagnoses for patients presenting with persistent febrile illnesses and highlights the critical need for timely intervention to improve outcomes.

## Case presentation

A 38-year-old previously healthy man presented with a three-week history of nighttime fever, accompanied by drenching sweats with no chills. He reported decreased appetite, a bitter taste in his mouth, and throat dryness, although he could swallow liquids and eat normally. There was no cough, hemoptysis, chest pain, shortness of breath, weight loss, diarrhea, constipation, stool color changes, or dysuria. In 2018, he experienced similar symptoms and sought care at a private hospital. At that time, all workups were negative, and he improved after one week of antibiotics. He had no chronic conditions and was not on any long-term medications.

On examination, his vital signs were as follows: temperature 38.6°C (tympanic), heart rate 128 bpm, respiratory rate 23 breaths/minute, blood pressure 131/81 mmHg, and SpO_2_ 100%. He weighed 85 kg. He appeared comfortable in bed, not in respiratory distress, but had pallor and dry mucous membranes. There was no lymphadenopathy. His chest exam revealed equal breath sounds bilaterally, without added sounds. The cardiovascular exam noted tachycardia with normal heart sounds and no murmurs. His abdomen was soft, non-tender, and without distention or organomegaly. Neurological examination was normal, with a Glasgow Coma Score (GCS) of 15/15.

Initial laboratory investigations revealed a white blood count (WBC) of 6.8 x 10^9^/L, hemoglobin of 6.4 g/dL, mean corpuscular volume (MCV) of 63.2 fL, mean corpuscular hemoglobin (MCH) of 21.4 pg, platelets of 85 x 10^9^/L, C-reactive protein (CRP) of 197.1 mg/L, and aspartate aminotransferase (AST) of 45 U/L (Table 1).

**Table 1:** Initial laboratory investigations EBV PCR: Epstein-Barr virus polymerase chain reaction; AST: aspartate aminotransferase; ALT: alanine aminotransferase; HbA1c: glycated hemoglobin A1c; CRP: C-reactive protein

Parameters	On Admission	5 Days After Admission	60 Days After Admission (4 Days Before Death)	63 Days After Admission (1 Day Before Death)	Reference Values
Serum ferritin (µg/L)	14.700	17.285	>100.000	>100.000	48-420
Serum triglycerides (mmol/L)	5.1	4.3	9.1	7.2	<1.7
EBV PCR (IU/mL)	66.700	-	336,165	8,151,750	Negative
Total leukocytes (×10^3^/uL)	4.5	5.9	0.8	1.9	6.2
Platelet (×10^3^/uL)	151	132	35	28	150-410
Hemoglobin (g/dL)	4.8	6.3	7.5	7.4	13-17
Serum urea (mmol/L)	4.2	5.5	9.9	8.5	2.5-7.8
Serum creatinine (µmol/L)	121	120	100	138	62-106
Serum potassium K (mmol/L)	4.5	3.6	4.2	4.3	3.5-5.3
Serum sodium (mmol/L)	133	132	128	121	133-146
Serum calcium (mmol/L)	2.44	2.39	2.44	2.56	2.2-2.6
Serum magnesium (mmol/L)	0.9	0.73	0.88	0.69	0.7-1
Alkaline phosphatase (IU/L)	86	72	120	145	40–129
AST (IU/L)	45	37	166	225	0-41
ALT (IU/L)	16	22	152	89	0-41
Serum total bilirubin (mg/dL)	22	23	74	44	0-21
Serum albumin (g/L)	29	19	14	13	35-50
Serum total protein (g/L)	72	56	41	36	60-80
Serum chloride (mmol/L)	100	99	102	95	95-108
Serum bicarbonate (mmol/L)	22	25	17	17	22-29
HbA1c (%)	5	-	-	-	<5.7
CRP (mg/L)	197	78	7.8	2.7	0-5
Procalcitonin (ng/mL)	15.1	2.8	-	32.8	<0.05

An abdominal ultrasound revealed hepatosplenomegaly and a well-defined hyperechoic lesion in the right liver lobe, suggestive of a hepatic hemangioma. He was admitted for further workup of unexplained fever and anemia. The next day, his condition deteriorated, and he was transferred to the ICU for closer monitoring. His WBC, platelet, and liver function tests began to deteriorate. He was initially treated for sepsis and started on empirical antibiotics (ceftriaxone 2 g once a day) while awaiting the results of the septic workup. Initial workups were negative, including full viral screening (negative for human immunodeficiency virus (HIV), cytomegalovirus (CMV), and hepatitis C virus (HCV)), tuberculosis, brucellosis, and bacterial cultures. Three sets of blood cultures also returned negative results.

**Figure 1:**
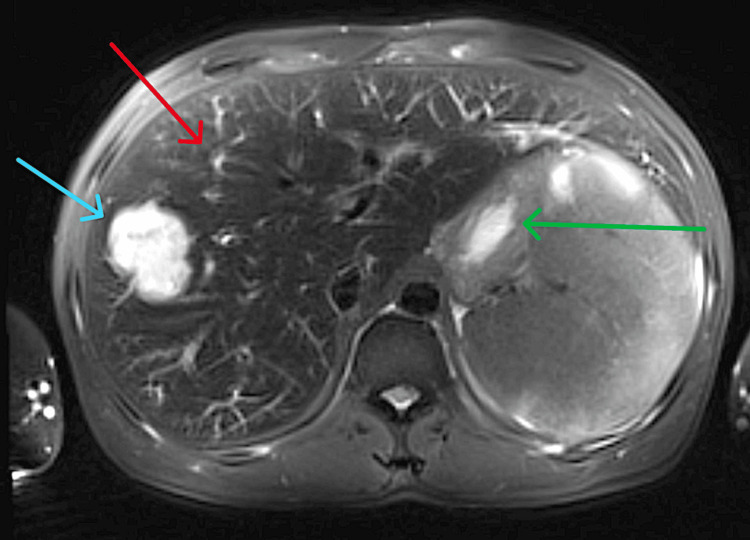
The MRI of the abdomen revealed hepatomegaly with hemosiderosis (red arrow), hepatic hemangioma (blue arrow), and splenomegaly with hemorrhagic infarcts (green arrow). MRI: magnetic resonance imaging

**Figure 2:**
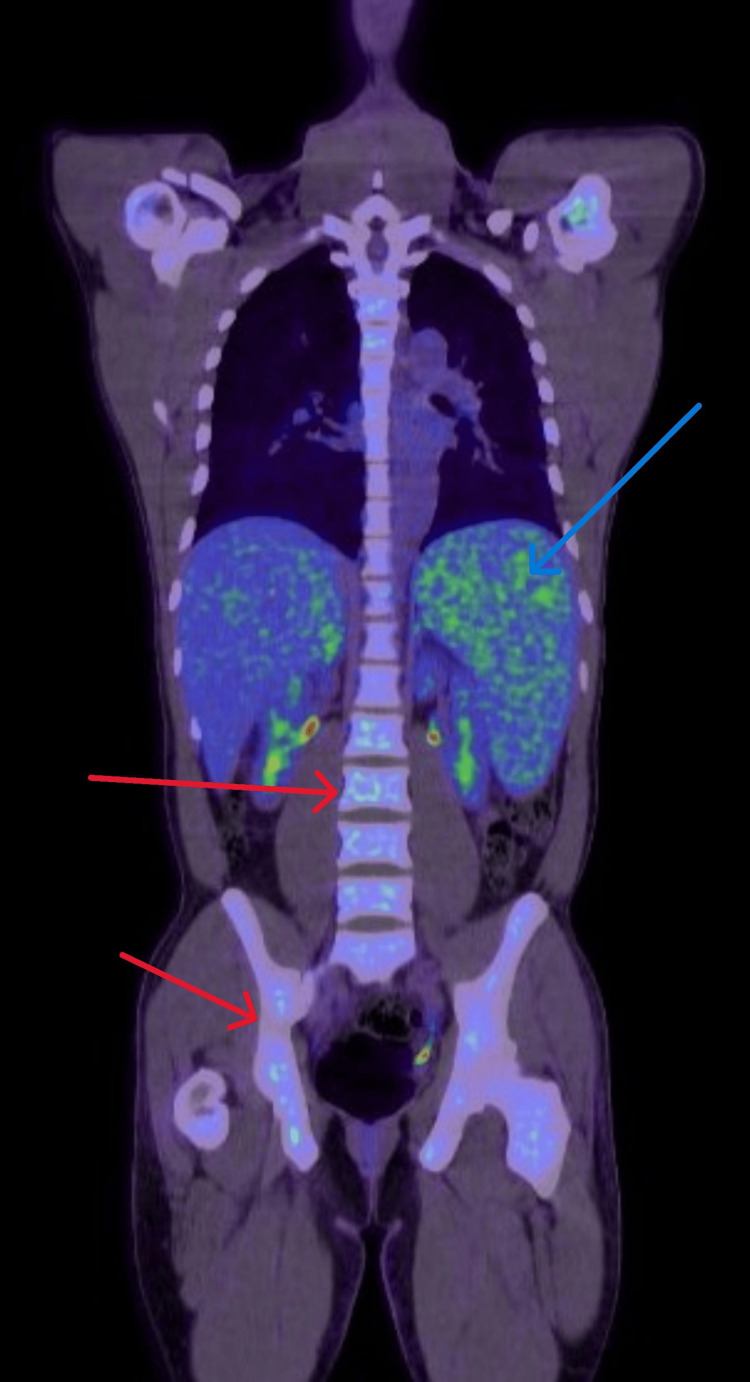
The PET scan revealed diffuse increased metabolic activity in the spleen (blue arrow) and diffuse bone marrow activation or infiltration (red arrows). PET scan: positron emission tomography scan

Further investigation showed markedly elevated ferritin levels (14,700, peaking at 100,000 μg/L), elevated triglycerides (5.4 mmol/L), and a highly positive EBV PCR (Table 1). Additional radiological imaging, including an abdominal magnetic resonance imaging (MRI) (Figure 1) and positron emission tomography (PET) scan, confirmed hepatosplenomegaly with diffuse hemosiderosis, a benign-appearing hepatic hemangioma, splenomegaly with ill-defined infiltrative lesions, two hemorrhagic infarcts, and diffuse bone marrow signal alterations (Figure 2).

A hematology consult was obtained, and a bone marrow biopsy showed hypercellular marrow with trilineage hematopoiesis, increased erythropoiesis, and evidence of hemophagocytosis. Based on these findings, he was diagnosed with HLH secondary to EBV infection. He was started on dexamethasone, broad-spectrum antibiotics, and multiple transfusions of packed red blood cells. He also received six doses of intravenous immunoglobulin (IVIG), followed by etoposide per the HLH protocol, and tocilizumab, rituximab, daily dexamethasone, and entecavir. Despite aggressive treatment, the patient did not respond well to the HLH protocol. His condition rapidly deteriorated, and after a sudden cardiac arrest, he could not be revived despite full CPR efforts. The patient passed away after a 64-day hospital stay.

## Discussion

HLH is a hyperinflammatory syndrome characterized by excessive immune activation, often leading to tissue destruction and multiorgan failure. The North American Consortium for Histiocytosis (NACHO) recommendations [1] distinguish between HLH syndrome, which involves pathological immune activation often linked to genetic defects, and HLH disease, where specific genetic or environmental causes are identified. This terminology helps clarify the confusion associated with the traditional labels of "primary" and "secondary" HLH, recognizing that both can be triggered by infections or immune-activating events, regardless of genetic predisposition.

The pathophysiology of HLH involves dysregulation of immune responses, predominantly driven by activated macrophages and natural killer (NK) cells. This activation of immune cells results in a cytokine storm characterized by elevated levels of interferon-gamma and other inflammatory cytokines. This excessive cytokine production is critical to the clinical severity of HLH and is associated with high mortality rates. While hemophagocytosis, the engulfment of host cells by activated macrophages, supports the diagnosis of HLH, it is not a requisite finding, as evidenced by the patient's presentation of cytopenias and elevated ferritin levels [2,7-9].

Infections, particularly those caused by viruses such as EBV, are common triggers of HLH. Primary EBV infection can provoke HLH, particularly in individuals with underlying defects in immune regulation, such as those with X-linked lymphoproliferative disease [2]. This case highlights the role of EBV in inciting a robust immune response leading to HLH, emphasizing the importance of vigilance in patients presenting with atypical manifestations following viral infections.

While this patient's HLH episode was linked to EBV, understanding its etiology requires understanding its genetic landscape. Many genetic defects associated with HLH impact the perforin-dependent cytotoxicity pathway, which is essential for regulating immune responses. Assessing genetic predisposition is vital, particularly in familial cases, as it informs prognosis and management strategies, including the potential need for hematopoietic cell transplantation (HCT) in cases of recurrence [10,11].

HLH typically presents as an acute or subacute febrile illness characterized by multiple organ involvement. Symptoms often mimic common infections, such as fever of unknown origin, hepatitis, or encephalitis. Some patients experience chronic "stuttering" presentations characterized by recurrent fevers of unknown origin and may fulfill only a subset of the classic diagnostic criteria [5]. HLH can be diagnosed if a mutation in a recognized causative gene or a patient meets at least five of the eight diagnostic criteria outlined in the HLH-2004 protocol [12].

Several key clinical findings were reported in a study involving 369 patients (HLH-2004) [12]. Fever was present in 95% of cases, with splenomegaly in 89%. Bicytopenia occurred in 92% of patients, while hypertriglyceridemia or hypofibrinogenemia was seen in 90% [12]. Hemophagocytosis was detected in 82% of cases, and 94% of patients had ferritin levels exceeding 500 µg/L. Additionally, 71% exhibited low or absent NK cell activity; elevated soluble CD25 was found in 97% [12].

Laboratory and radiographic findings in HLH frequently show cytopenias, particularly anemia and thrombocytopenia, occurring in over 80% of patients. Platelet counts range from 3,000 to 292,000/µL, with a median of 69,000, and hemoglobin levels average approximately 7.2 g/dL [13,14]. High serum ferritin levels are standard, providing diagnostic sensitivity and specificity, with median ferritin reported at 2,950 ng/mL in the HLH-94 study [13,15]. Nearly all patients present with liver function abnormalities, including elevated AST, ALT, gamma-glutamyl transferase (GGT), and bilirubin levels, with 50-90% having liver enzyme levels exceeding three times the standard limit [16]. Hypertriglyceridemia and coagulation abnormalities, such as elevated D-dimer, are also common, often due to liver dysfunction and disseminated intravascular coagulation.

Neurologic abnormalities occur in approximately one-third of HLH patients, with symptoms such as seizures, mental status changes, and ataxia, which may dominate the clinical picture or appear before other HLH signs [17,18]. HLH can also impact the respiratory, renal, and integumentary systems. Respiratory complications, including acute respiratory distress syndrome, may require ventilatory support. Renal dysfunction is common and can present with hyponatremia, possibly due to the syndrome of inappropriate ADH (SIADH). Skin manifestations vary widely, from rashes and edema to petechiae and purpura, often accompanied by bleeding due to coagulation issues, liver failure, or thrombocytopenia [19].

HLH is often associated with viral infections, particularly EBV, CMV, and other viruses [19,20]. An HLH-like syndrome has been reported in association with SARS-CoV-2 (the virus responsible for COVID-19) [21]. Furthermore, HLH can occur in conjunction with malignancies, most commonly lymphoid cancers, leukemias, and solid tumors [22]. Lastly, HLH has been observed in patients with inherited immunodeficiency disorders, including those with genetic mutations known to be associated with HLH [23].

Patients with HLH require intensive monitoring and supportive care to manage organ dysfunction, bleeding, and infections. Transfusions are commonly used for cytopenias, with higher thresholds for platelet transfusions due to the increased risk of bleeding. Coagulation abnormalities and bleeding are treated with plasma products, while infections are managed through prophylaxis and prompt use of broad-spectrum antibiotics. Disease-specific markers such as ferritin and cytokine levels are monitored throughout treatment to assess response. For patients who do not fully respond to initial therapy, HCT is often necessary, and long-term follow-up is essential to prevent relapses and manage post-treatment complications [6,24].

The prognosis for HLH is poor without treatment, particularly for those with inherited HLH gene mutations, who may survive only about two months. In treated patients, survival rates vary; a study of 162 adults showed a 58% overall survival rate, with better outcomes for those undergoing allogeneic HCT, achieving a five-year survival rate of 66% if in remission at transplant. Higher serum ferritin levels and the presence of malignancies are associated with worse outcomes, and most relapses occur within the first year, particularly in patients with HLH gene mutations. Careful management of underlying conditions and avoidance of triggers is crucial for reducing relapse risk, with advancements in early diagnosis and HCT techniques expected to improve survival rates [13,25,26].

## Conclusions

This case underscores the need for clinicians to recognize HLH as a serious complication of EBV infection. Due to HLH's rapid onset and severity, prompt diagnosis and intervention are crucial. The report highlights the connection between viral infections and hyperinflammatory responses and urges awareness of atypical symptoms after EBV infection. It also calls for more research into genetic factors related to HLH to enhance treatment and prognostic strategies. It emphasizes the complexities of managing HLH and the importance of clinical vigilance and collaboration.
